# Conditional dependency of oncogenic KRAS in driving ketone body catabolism and pancreatic cancer growth

**DOI:** 10.1016/j.jbc.2026.113332

**Published:** 2026-07-14

**Authors:** Runze Wang, Xue Li, Tongxin Zhang, Yinuo Chen, Juanjuan Liu, Yuanjing Wang, Xuanzhe Wang, Lianjing Cao, Zhimin Lu, Leina Ma

**Affiliations:** 1 Department of Pharmaceutics, School of Pharmacy, Qingdao University, Qingdao, China; 2 Department of Oncology, Cancer Institute, The Affiliated Hospital of Qingdao University, Qingdao University, Qingdao Cancer Institute, Qingdao, Shandong, China; 3 Pancreatic Disease Institute, The Affiliated Hospital of Qingdao University, Qingdao, China; 4 Shandong Provincial Key Laboratory of Clinical Research for Pancreatic Diseases, The Affiliated Hospital of Qingdao University, Qingdao, China; 5 Zhejiang Key Laboratory of Pancreatic Disease, The First Affiliated Hospital, Zhejiang Key Laboratory of Frontier Medical Research on Cancer Metabolism, Institute of Translational Medicine, Zhejiang University School of Medicine, Hangzhou, Zhejiang, China; 6 Institute of Fundamental and Transdisciplinary Research, Zhejiang University Cancer Center, Zhejiang University, Hangzhou, Zhejiang, China; 7 The Center for Translational Research, The Second Affiliated Hospital and Institute of Translational Medicine, Zhejiang University, Hangzhou, Zhejiang, China; 8 Cancer Institute, The Affiliated Hospital of Qingdao University, Qingdao University, Qingdao Cancer Institute, Qingdao, Shandong, China

**Keywords:** ketolysis, metabolic reprogramming, oncogenic KRAS, PDAC, tumor microenvironment

## Abstract

Pancreatic ductal adenocarcinoma (PDAC) is a highly lethal malignancy driven predominantly by oncogenic KRAS mutations, which enforce extensive metabolic reprogramming to support tumor progression. Although ketone body catabolism has emerged as a critical metabolic adaptation in PDAC, the signaling mechanisms that link mutant KRAS to the ketolytic machinery remain largely unexplored. Here, we identify a previously unrecognized, context-dependent role of oncogenic KRAS in driving ketone body utilization. We show that KRAS mutation alone is insufficient to fully activate ketolysis; instead, it primes the ketolytic pathway in a manner that requires cooperative input from additional tumor microenvironment signals. Mechanistically, oncogenic KRAS engages a downstream signaling cascade that leads to specific post-translational modifications of key mitochondrial enzymes. These modifications enhance the flux of ketone body catabolism, thereby increasing acetyl-CoA and ATP production and promoting pancreatic cancer cell proliferation and xenograft tumor growth. In a clinical PDAC cohort, activation of this KRAS-dependent ketolytic axis was elevated in KRAS-mutant tumors compared with KRAS wild-type cases, albeit with a trend that requires further validation. Collectively, our findings define a conditional dependency of mutant KRAS on cooperative signals to drive ketone body catabolism, linking oncogenic signaling to mitochondrial ketone metabolism in PDAC. This study expands our understanding of KRAS-driven metabolic reprogramming and highlights the ketolytic pathway as a context-dependent vulnerability for therapeutic intervention in pancreatic cancer.

Pancreatic ductal adenocarcinoma (PDAC) remains one of the most aggressive and lethal human malignancies, with an extremely low 5-year survival rate and limited therapeutic options (1). A hallmark of PDAC is the presence of oncogenic KRAS mutations in more than 90% of cases, which drive sustained proliferation, metastasis, therapy resistance, and profound metabolic reprogramming. In the tumor microenvironment, additional signaling inputs—such as those from growth factors and cytokines—are known to cooperate with mutant KRAS to support tumor development and progression. However, the molecular mechanisms by which KRAS integrates with these cooperative signals to regulate metabolic adaptation, particularly ketone body metabolism, remain largely undefined.

Altered metabolism is now recognized as a core hallmark of cancer. To sustain rapid proliferation and survive in the nutrient-poor, hypoxic tumor microenvironment, PDAC cells undergo dramatic metabolic rewiring, including enhanced utilization of alternative energy sources such as ketone bodies (2, 3, 4). Ketone bodies, including β-hydroxybutyrate (β-HB) and acetoacetate (AcAc), are efficiently catabolized through the ketolytic pathway to generate acetyl-CoA and ATP, thereby supporting tumor growth and survival (5, 6, 7). The rate-limiting enzyme of ketolysis has been shown to be upregulated in multiple cancer types, including PDAC, and its expression correlates with poor prognosis (8, 9). Despite the established role of ketone body catabolism in cancer progression, how the ketolytic machinery is dynamically regulated at the post-translational level and linked to major oncogenic signals in PDAC remains poorly understood.

Emerging evidence suggests that the metabolic functions of oncogenic KRAS are not always cell-autonomous or constitutive; instead, they often depend on cooperative inputs from the tumor microenvironment. This context-dependent nature of KRAS-driven metabolism is particularly relevant for alternative fuel utilization, such as ketone body catabolism, where the full activation of the ketolytic pathway may require additional signals beyond KRAS mutation alone (10).

In this study, we hypothesized that oncogenic KRAS primes the ketolytic machinery, but its full activation depends on cooperative microenvironmental cues, such as IGF1 signaling (11). To test this, we focused on two key mitochondrial enzymes: SUCLA2, a TCA cycle enzyme that generates succinyl-CoA, and OXCT1, the rate-limiting enzyme of ketolysis. Notably, succinyl-CoA is not only a metabolic intermediate but also a substrate for protein succinylation, a post-translational modification. PIN1, a prolyl isomerase that recognizes phosphorylated Ser/Thr-Pro motifs, was also investigated as a potential conformational regulator (2).

Here, we demonstrate that mutant KRAS alone is insufficient to fully activate ketolysis; rather, it requires cooperative input from IGF1 to engage an ERK-PIN1-SUCLA2 axis. Mechanistically, ERK phosphorylates SUCLA2 at S124, creating a PIN1-binding motif. PIN1-catalyzed cis-trans isomerization of phospho-SUCLA2 promotes its interaction with OXCT1, enabling SUCLA2 to succinylate OXCT1 at K421. This modification enhances ketolytic flux, elevates acetyl-CoA and ATP production, and accelerates PDAC cell proliferation and tumor growth. Clinically, activation of this axis shows an elevated trend in KRAS-mutant PDAC tissues. Collectively, our findings uncover a conditional dependency of mutant KRAS on cooperative signals to drive ketone body catabolism, linking oncogenic signaling to mitochondrial ketone metabolism in PDAC and providing a promising therapeutic target for this devastating disease.

## Results

### Oncogenic KRAS and IGF1 converge on ERK to induce SUCLA2 phosphorylation and SUCLA2–OXCT1 complex formation

**Figure 1:**
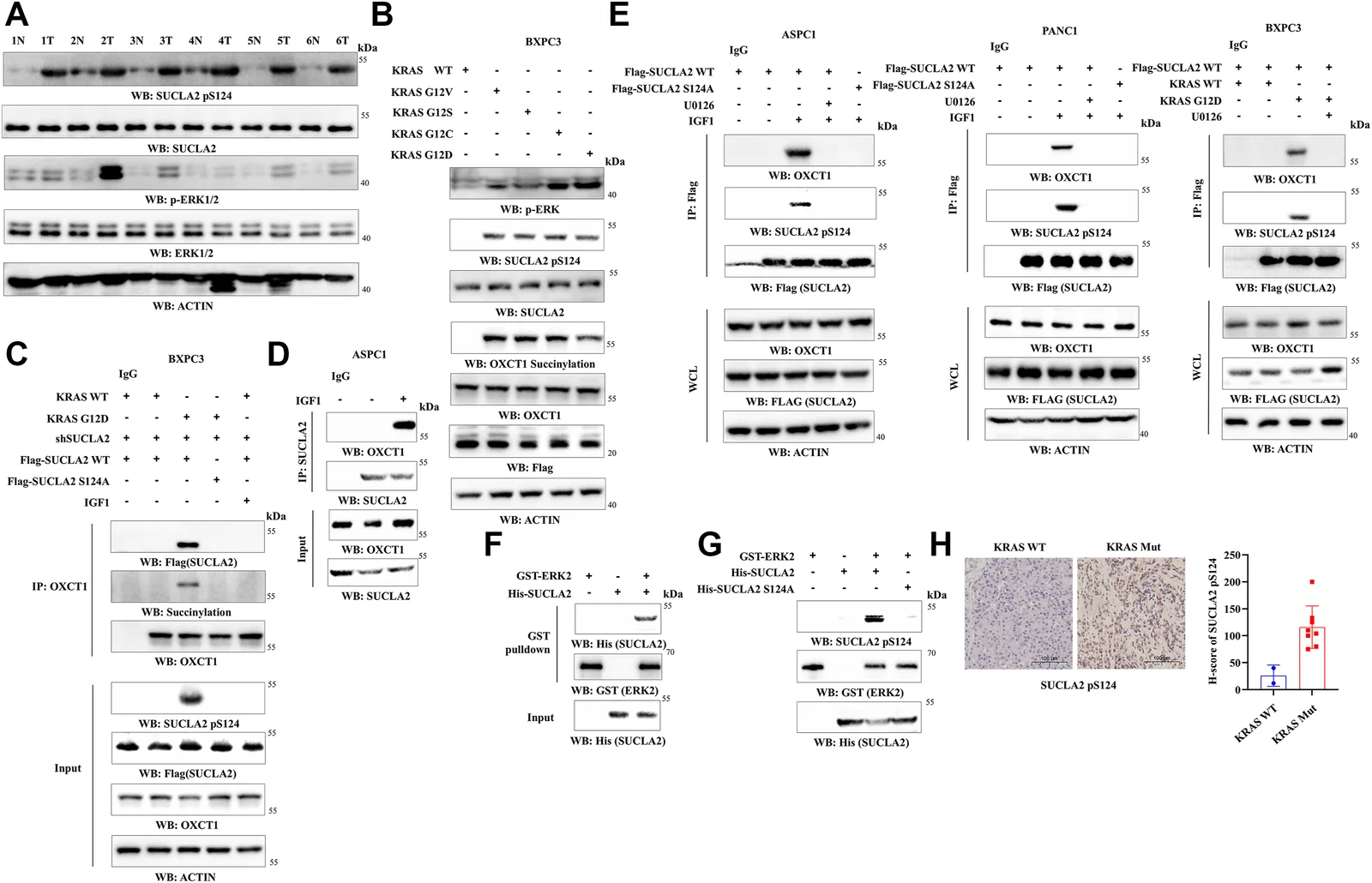
**Oncogenic KRAS and IGF1 converge on ERK to induce SUCLA2 phosphorylation and SUCLA2–OXCT1 complex formation.***A*, Immunoblot analysis of six paired human pancreatic cancer (T) and adjacent non-tumor (N) tissues using the indicated antibodies. *B*, BXPC3 cells expressing KRAS WT or the indicated KRAS mutants were lysed and analyzed by immunoblotting with the indicated antibodies. *C*, BXPC3 cells expressing KRAS WT or KRAS G12D together with Flag-SUCLA2 WT or Flag-SUCLA2 S124A were subjected to co-immunoprecipitation (co-IP) and immunoblot analysis. *D*, ASPC1 cells treated with or without IGF1 (6 h) were analyzed by co-IP and immunoblotting. *E*, ASPC1 and PANC1 cells expressing Flag-SUCLA2 WT or S124A were treated with IGF1 (6 h) with or without U0126 pretreatment (30 min). BXPC3 cells expressing KRAS WT or G12D together with Flag-SUCLA2 WT were treated with U0126 (6 h). Lysates were analyzed by co-IP and immunoblotting. *F*, GST pull-down assay using purified recombinant GST-ERK2 and His-SUCLA2 proteins. *G*, *in vitro* kinase assay with active GST-ERK2 and His-SUCLA2 or His-SUCLA2 S124A, followed by immunoblotting. *H*, immunohistochemical staining of human PDAC samples (2 KRAS WT, 8 KRAS Mut) with an anti-SUCLA2 pS124 antibody. *Right*: quantification of SUCLA2 pS124 levels (n = 10).

To identify downstream effectors of KRAS/IGF1 signaling that might regulate ketolysis, we examined TCA cycle enzymes known to interact with ketolytic proteins. SUCLA2 emerged as a candidate because it produces succinyl-CoA, a substrate both for the TCA cycle and for protein succinylation. To assess the clinical relevance of the ERK–SUCLA2 axis in PDAC, we first performed immunoblot analysis on six pairs of human pancreatic tumor and adjacent non-tumor tissues. Phosphorylation of both ERK and SUCLA2 at S124 was markedly increased in tumor tissues compared with matched normal controls (Fig. 1*A*). We next examined upstream regulators of this axis in PDAC cells. Ectopic expression of various oncogenic KRAS mutants (G12V, G12S, G12C, and G12D) in KRAS wild-type BXPC3 cells strongly enhanced ERK and SUCLA2 S124 phosphorylation as well as OXCT1 succinylation (Fig. 1*B*). Notably, overexpression of KRAS G12D, but not KRAS WT, promoted the interaction between exogenous SUCLA2 and endogenous OXCT1, and increased OXCT1 succinylation; these effects were completely abolished by the SUCLA2 S124A mutation. Interestingly, IGF1 treatment alone failed to induce these changes in BXPC3 cells (Fig. 1*C*). In ASPC1 cells, IGF1 stimulation similarly induced SUCLA2 S124 phosphorylation and SUCLA2–OXCT1 binding (Fig. 1*D*). Both responses were blocked by pretreatment with the ERK inhibitor U0126 in ASPC1 and PANC1 cells. Likewise, U0126 pretreatment eliminated KRAS G12D-induced SUCLA2 phosphorylation and SUCLA2–OXCT1 interaction (Fig. 1*E*). GST pull-down and *in vitro* kinase assays confirmed that ERK2 directly binds to and phosphorylates SUCLA2 at S124 (Fig. 1, *F* and *G*). Importantly, immunohistochemical analysis of clinical PDAC specimens exhibited a trend toward higher SUCLA2 pS124 expression in KRAS-mutant tumors than in KRAS wild-type tumors (Fig. 1*H*). Collectively, these results demonstrate that both oncogenic KRAS and IGF1 signaling act through ERK to trigger SUCLA2 phosphorylation and promote SUCLA2–OXCT1 complex assembly.

### PIN1-mediated cis-trans isomerization of phosphorylated SUCLA2 is required for OXCT1 binding

**Figure 2:**
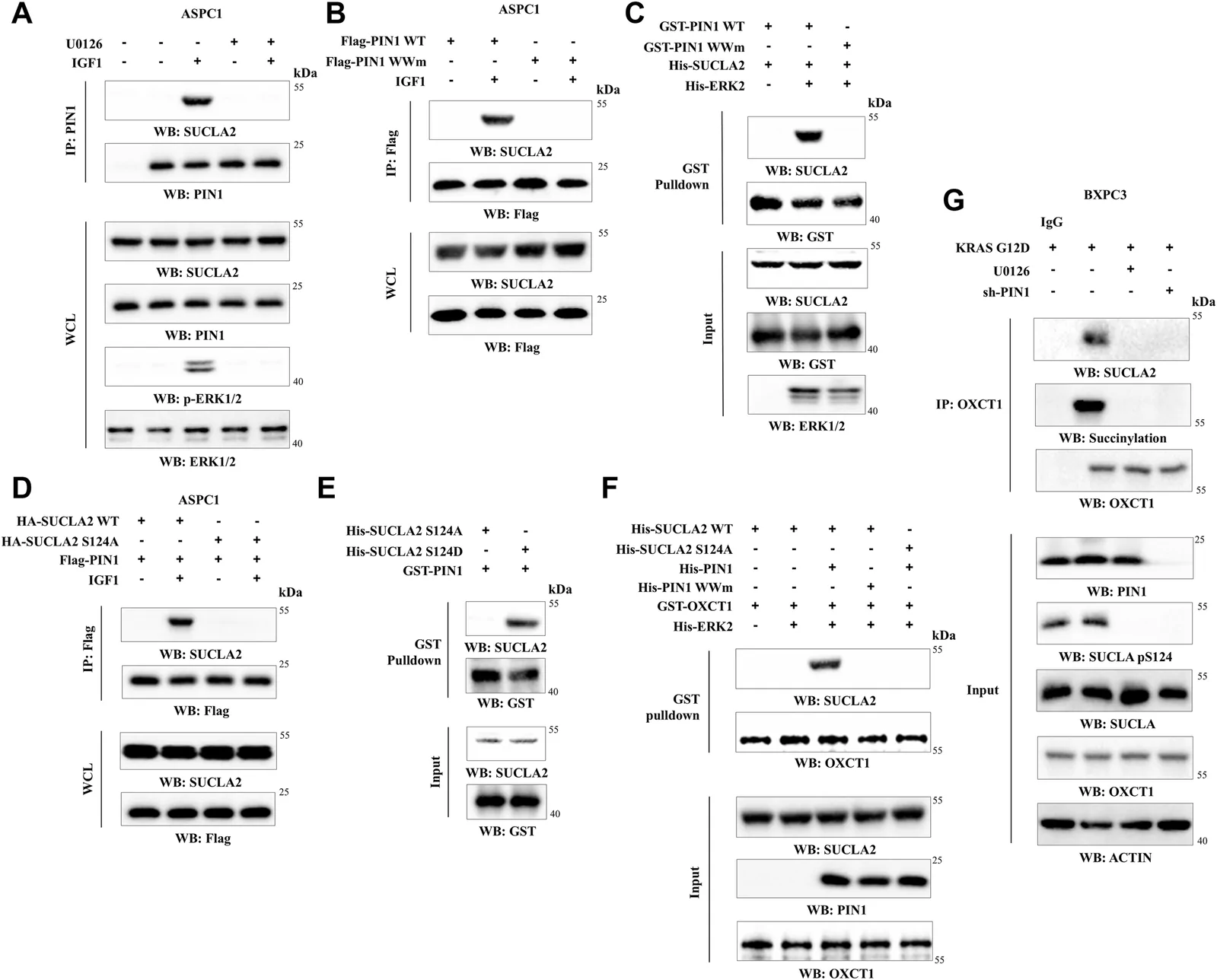
**PIN1-mediated cis-trans isomerization of phosphorylated SUCLA2 is required for OXCT1 binding.***A*, ASPC1 cells treated with or without IGF1 (6 h) with U0126 pretreatment where indicated were analyzed by co-IP and immunoblotting. *B*, ASPC1 cells expressing Flag-PIN1 WT or WW domain mutant (WWm) were treated with IGF1 (6 h) and analyzed by co-IP. *C*, GST pull-down assay using purified GST-PIN1 WT or WWm, His-SUCLA2, and active ERK2 as indicated. *D*, ASPC1 cells co-expressing HA-SUCLA2 WT or S124A with Flag-PIN1 WT were treated with IGF1 (6 h) and analyzed by co-IP. *E*, GST pull-down assay using GST-PIN1 and His-SUCLA2 S124A or S124D. *F*, *in vitro* reconstitution of SUCLA2–OXCT1 binding using purified GST-OXCT1, His-PIN1 WT or WWm, and His-SUCLA2 WT or S124A, in the presence or absence of active ERK2, followed by GST pull-down. *G*, BXPC3 cells expressing KRAS G12D were treated with U0126 (2 h) or transduced with PIN1 shRNA, followed by co-IP and immunoblotting.

Given that SUCLA2 contains a conserved Ser-Pro motif (S124) that matches the recognition sequence of the prolyl isomerase PIN1—a known regulator of oncogenic signaling—we asked whether PIN1 interacts with phosphorylated SUCLA2. Protein interacting with never in mitosis A1 (PIN1) specifically recognizes phosphorylated Ser/Thr–Pro motifs and catalyzes cis–trans isomerization of substrate proteins, thereby modulating their conformation and function (12, 13, 14). To test whether PIN1 interacts with phosphorylated SUCLA2, we performed Co-immunoprecipitation analyses. IGF1 treatment robustly enhanced the association between SUCLA2 and PIN1, which was blocked by U0126 (Fig. 2*A*). The PIN1 WW domain was essential for this interaction, as a WW domain mutant failed to bind phosphorylated SUCLA2 (Fig. 2, *B* and *C*). The SUCLA2 S124A mutation abrogated PIN1 binding, whereas the phosphomimetic S124D variant directly interacted with PIN1 (Fig. 2, *D* and *E*). *In vitro* reconstitution assays further showed that stable SUCLA2–OXCT1 binding required wild-type PIN1, whereas the WW domain mutant failed to support binding. (Fig. 2*F*). Moreover, KRAS G12D-induced SUCLA2–OXCT1 association was markedly reduced by U0126 treatment or PIN1 knockdown (Fig. 2*G*). Together, these data indicate that ERK-mediated phosphorylation of SUCLA2 at S124 recruits PIN1 to catalyze cis-trans isomerization, a critical step for SUCLA2–OXCT1 complex formation.

### SUCLA2 mediates site-specific succinylation of OXCT1 at K421 upon KRAS activation

**Figure 3:**
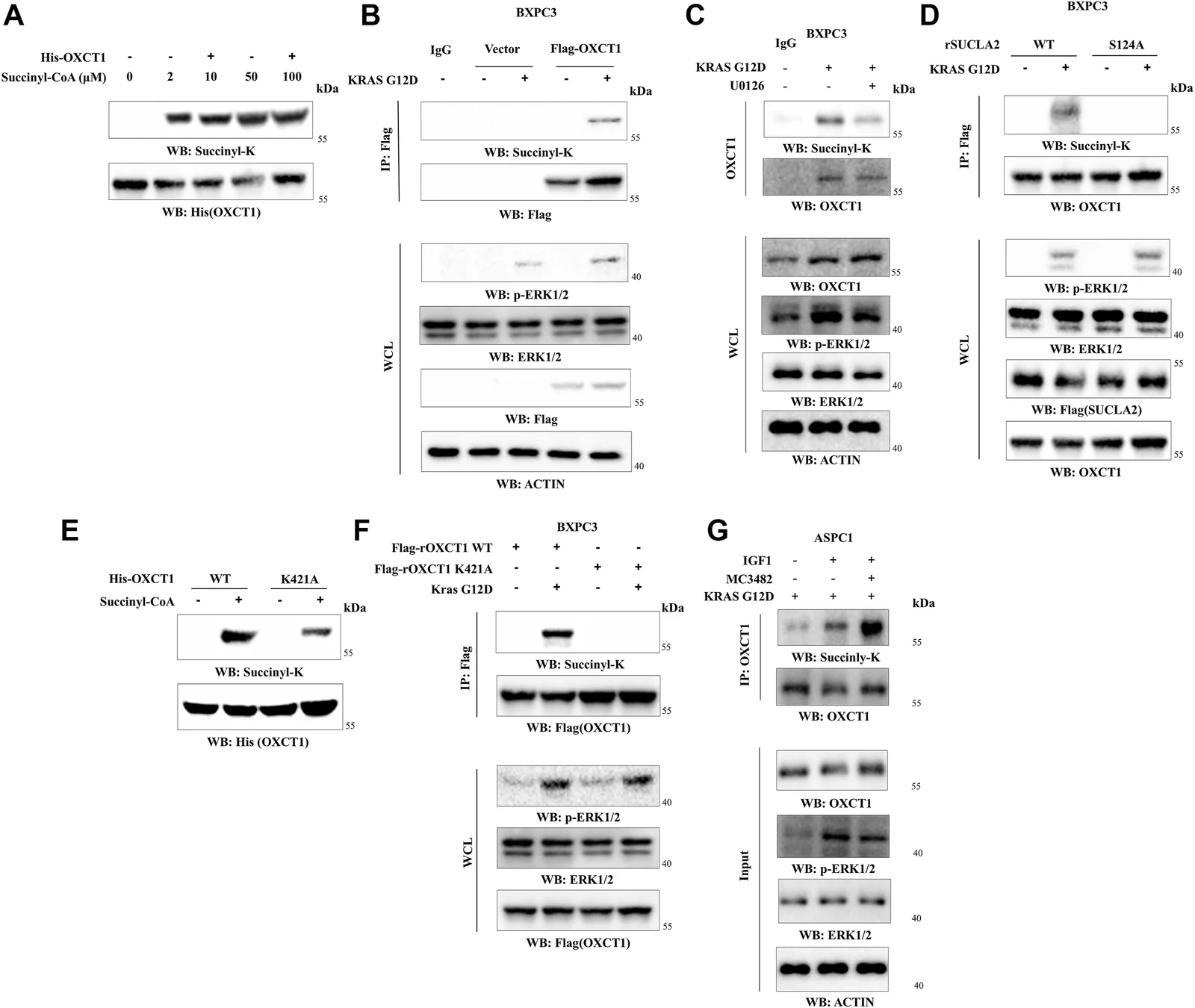
**SUCLA2 mediates site-specific succinylation of OXCT1 at K421 upon KRAS activation.***A*, purified GST-OXCT1 was incubated with increasing concentrations of succinyl-CoA and immunoblotted with an anti-succinyl-lysine (succinyl-K) antibody. *B*, BXPC3 cells overexpressing KRAS G12D with Flag-OXCT1 or empty vector were analyzed by co-IP and immunoblotting. *C*, BXPC3 cells expressing KRAS G12D were treated with or without U0126, and OXCT1 succinylation was assessed by co-IP. *D*, BXPC3 cells expressing KRAS G12D and reconstituted with SUCLA2 WT or S124A were analyzed by co-IP and immunoblotting. *E*, immunoblot analysis of purified GST-OXCT1 or GST-OXCT1 K421A after incubation with 10 mM succinyl-CoA. *F*, BXPC3 cells expressing KRAS G12D together with Flag-OXCT1 WT or K421A were analyzed by co-IP and immunoblotting. *G*, ASPC1 cells were treated with IGF1 in the presence of MC3482 (50 μm, 24 h). OXCT1 succinylation was analyzed by immunoprecipitation with anti-OXCT1 followed by immunoblotting with anti-succinyl-lysine.

Because SUCLA2 generates succinyl-CoA, and succinyl-CoA can succinylate lysine residues on target proteins, we tested whether OXCT1, the rate-limiting ketolytic enzyme, undergoes succinylation in a SUCLA2-dependent manner. SUCLA2 is a TCA cycle enzyme that reversibly converts succinate and CoA to succinyl-CoA (15, 16). To determine whether OXCT1 undergoes succinylation, we purified recombinant OXCT1 and performed an *in vitro* succinylation assay. OXCT1 was succinylated by succinyl-CoA in a dose-dependent manner (Fig. 3*A*). Consistently, KRAS activation induced succinylation of overexpressed OXCT1 in BXPC3 cells (Fig. 3*B*). To examine whether this modification depends on upstream ERK signaling, we treated KRAS-activated BXPC3 cells with the ERK inhibitor U0126. U0126 treatment markedly reduced OXCT1 succinylation (Fig. 3*C*), indicating that the ERK cascade is required for KRAS-induced OXCT1 succinylation.

We next asked whether SUCLA2 is directly responsible for OXCT1 succinylation. Co-immunoprecipitation experiments showed that wild-type (WT) SUCLA2 promoted OXCT1 succinylation, whereas the phosphorylation-deficient S124A mutant, which fails to bind OXCT1, did not (Fig. 3*D*). Our previous work identified lysine 421 (K421) as the major succinylation site on OXCT1 (17). *In vitro*, the OXCT1 K421A mutant showed markedly reduced succinylation compared with WT OXCT1 in the presence of succinyl-CoA (Fig. 3*E*). Similarly, KRAS activation-induced succinylation was completely abrogated in OXCT1 K421A-expressing BXPC3 cells (Fig. 3*F*). These results demonstrate that KRAS activation promotes SUCLA2 binding to OXCT1 and succinyl-CoA production, leading to OXCT1 succinylation at K421. To gain insight into the dynamic regulation of OXCT1 succinylation, we next sought to identify potential desuccinylases that might reverse this modification. Based on the subcellular localization of OXCT1 in the mitochondrial matrix and an extensive literature review, we speculated that SIRT5 is the most likely candidate desuccinylase for OXCT1. This hypothesis is supported by the following: (1) SIRT5 is the principal NAD^+^-dependent lysine desuccinylase localized in the mitochondrial matrix; (2) SIRT5 is known to regulate key enzymes in ketone body synthesis (*e*.*g*., HMGCS2) *via* desuccinylation; (3) proteomic studies have identified multiple metabolic enzymes, including TCA cycle and fatty acid oxidation enzymes, as SIRT5 substrates. Given that OXCT1 functions as a gatekeeper in ketone body utilization, it would be a logical target for SIRT5-mediated regulation. To test this hypothesis, we used two small-molecule compounds: MC3482 (a reported SIRT5 inhibitor) (18, 19). Treatment of ASPC1 cells with IGF1 in the presence of MC3482 revealed that MC3482 increased IGF1-induced OXCT1 succinylation.

### OXCT1 succinylation at K421 enhances ketolysis and ATP production in PDAC cells

**Figure 4:**
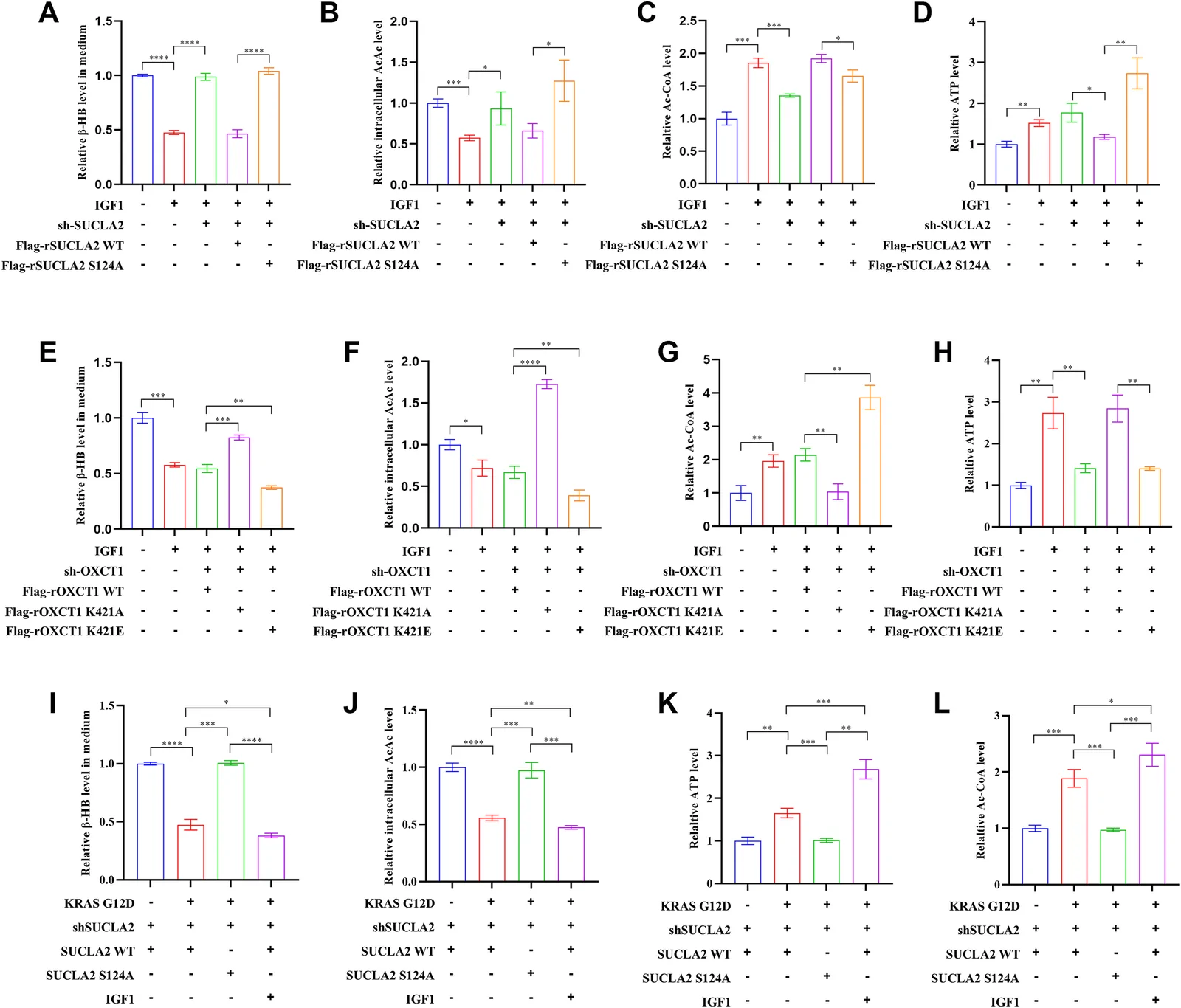
**OXCT1 succinylation at K421 enhances ketolysis and ATP production in PDAC cells.***A–D*, ASPC1 cells (control, SUCLA2 knockdown, or reconstituted with SUCLA2 WT or S124A) were cultured with 2 mM β-HB ± IGF1 for 24 h. Medium β-HB (*A*) and intracellular AcAc (*B*), acetyl-CoA (*C*), and ATP (*D*) were measured. *E–H*, ASPC1 cells (control, OXCT1 knockdown, or reconstituted with OXCT1 WT, K421A, or K421E) were treated as above. β-HB consumption (*E*), AcAc (*F*), acetyl-CoA (*G*), and ATP (*H*) were measured. *I–L*, BXPC3 cells (control, KRAS G12D overexpression, SUCLA2 knockdown, or reconstituted with SUCLA2 WT or S124A) were treated with 2 mM β-HB ± IGF1 for 24 h. β-HB (*I*), AcAc (*J*), acetyl-CoA (*K*), and ATP (*L*) were quantified. Data are mean ± SD of three independent experiments. ∗*p* < 0.05; ∗∗*p* < 0.01; ∗∗∗*p* < 0.001; ∗∗∗∗*p* < 0.0001.

To determine the functional significance of OXCT1 succinylation, we examined its impact on ketolytic flux and energy production, as enhanced ketolysis is known to support cancer cell survival under metabolic stress. To dissect the functional consequences of OXCT1 succinylation, we reconstituted WT SUCLA2, SUCLA2 S124A, WT OXCT1, OXCT1 K421A, or the succinylation-mimetic OXCT1 K421E in ASPC1 cells. After IGF1 stimulation, cells were cultured with β-hydroxybutyrate (β-HB). IGF1 treatment increased β-HB consumption (Fig. 4*A*), decreased acetoacetate (AcAc) accumulation (Fig. 4*B*), and elevated intracellular acetyl-CoA (Fig. 4*C*) and ATP levels (Fig. 4*D*). Knockdown of SUCLA2 (Fig. 4, *A*–*D*) or OXCT1 (Fig. 4, *E*–*H*) suppressed ketolysis, acetyl-CoA production, and ATP generation. These defects were rescued by re-expression of WT SUCLA2 (Fig. 4, *A*–*D*) or WT OXCT1 (Fig. 4, *E*–*H*), but not by the SUCLA2 S124A or OXCT1 K421A mutants. Notably, the constitutive succinylation mimic OXCT1 K421E further enhanced β-HB utilization (Fig. 4*E*), reduced AcAc (Fig. 4*F*), and increased acetyl-CoA (Fig. 4*G*) and ATP levels (Fig. 4*H*) compared with WT OXCT1.

We next examined whether KRAS activation promotes ketone body utilization. BXPC3 cells reconstituted with WT SUCLA2 or S124A and overexpressing KRAS G12D were analyzed. KRAS activation enhanced β-HB consumption (Fig. 4*I*), decreased AcAc (Fig. 4*J*), and increased acetyl-CoA (Fig. 4*K*) and ATP levels (Fig. 4*L*). Moreover, combined IGF1 and KRAS G12D stimulation further promoted ketolysis. Collectively, these findings indicate that IGF1 cooperates with KRAS to induce OXCT1 K421 succinylation, thereby enhancing ketolysis and energy production in PDAC cells.

### OXCT1 succinylation at K421 promotes PDAC cell proliferation and tumor growth

**Figure 5:**
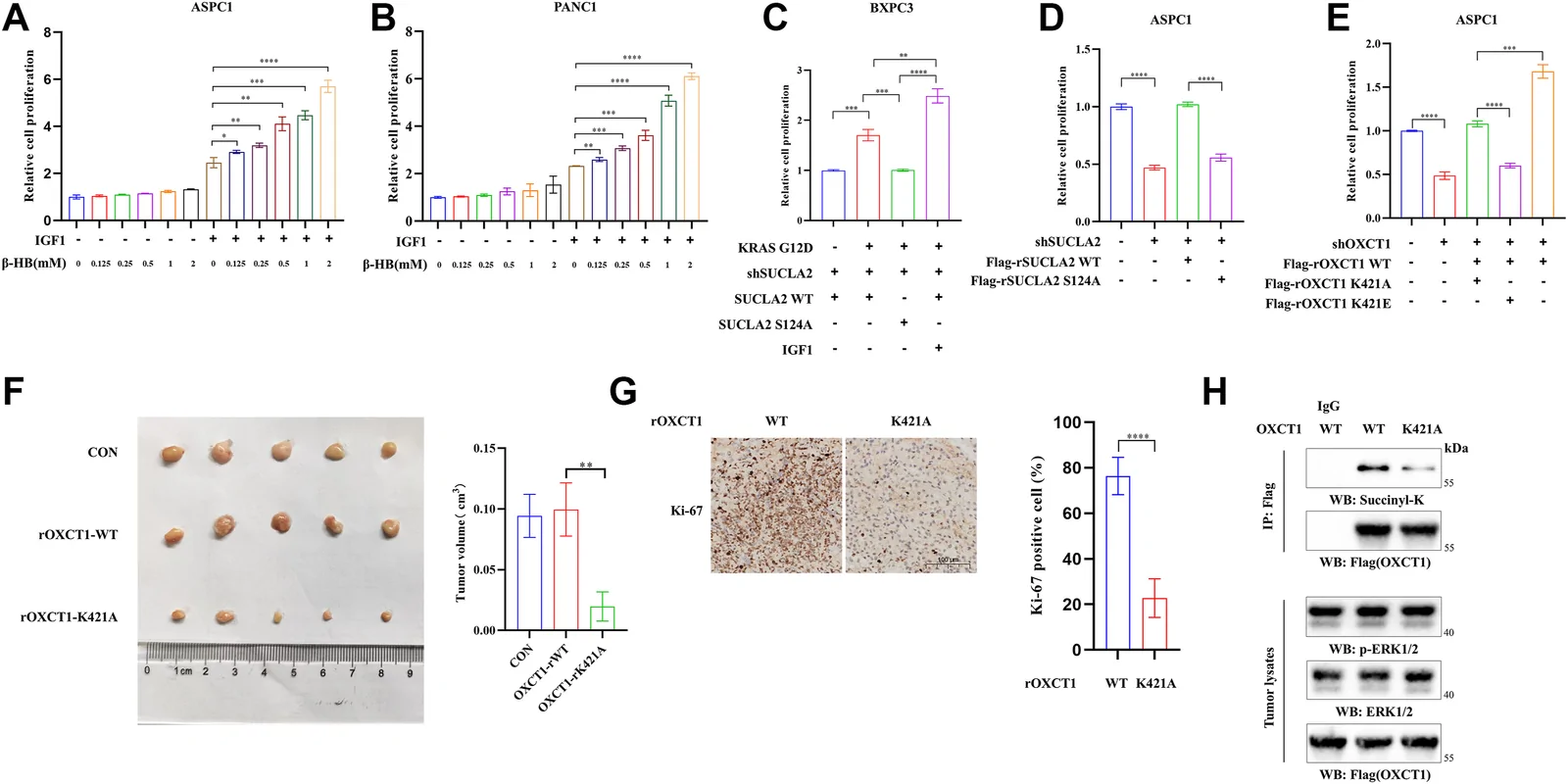
**OXCT1 succinylation at K421 promotes PDAC cell proliferation and tumor growth.***A* and *B*, ASPC1 (*A*) and PANC1 (*B*) cells were treated with the indicated concentrations of β-HB ± IGF1 for 48 h, and cell proliferation was measured. *C*, BXPC3 cells (control, KRAS G12D, SUCLA2 knockdown, or reconstituted with SUCLA2 WT or S124A) were cultured with 2 mM β-HB ± IGF1 for 48 h, and proliferation was assessed. *D* and *E*,) ASPC1 cells with SUCLA2 (*D*) or OXCT1 (*E*) knockdown were reconstituted with the indicated constructs and cultured with β-HB + IGF1 for 48 h. Proliferation was measured. *F–H*, ASPC1 cells expressing control shRNA, shRNA-resistant rOXCT1 WT, or rOXCT1 K421A were injected subcutaneously into nude mice (n = 5 per group). Tumors were harvested at day 21. Tumor volumes (*F*), Ki-67 immunohistochemistry (*G*), and OXCT1 succinylation by co-IP (*H*) are shown. Data are mean ± SD of three independent experiments. ∗*p* < 0.05; ∗∗*p* < 0.01; ∗∗∗*p* < 0.001; ∗∗∗∗*p* < 0.0001.

Having established that OXCT1 K421 succinylation promotes ketolysis and ATP production, we next asked whether this translates into increased PDAC cell proliferation and tumor growth. ASPC1 and PANC1 cells were treated with increasing concentrations of β-HB with or without IGF1. Under IGF1 stimulation, β-HB promoted cell proliferation in a concentration-dependent manner (Fig. 5, *A* and *B*). In BXPC3 cells, KRAS G12D overexpression enhanced proliferation in the presence of β-HB, and IGF1 further synergized with KRAS G12D to increase proliferation (Fig. 5*C*). Knockdown of SUCLA2 or OXCT1 attenuated IGF1-induced proliferation in ASPC1 cells; this effect was rescued by re-expression of WT SUCLA2 (Fig. 5*D*) or WT OXCT1 (Fig. 5*E*), but not by the SUCLA2 S124A or OXCT1 K421A mutants.

To assess the role of OXCT1 K421 succinylation *in vivo*, we established ASPC1 cells stably expressing WT OXCT1 or the non-succinylatable K421A mutant and generated subcutaneous xenografts in nude mice. Tumors expressing OXCT1 K421A exhibited significantly reduced growth (Fig. 5*F*) and lower Ki-67 positivity (Fig. 5*G*). Immunoprecipitation of xenograft lysates confirmed that OXCT1 succinylation was markedly diminished in K421A tumors (Fig. 5*H*). These results demonstrate that OXCT1 succinylation at K421 enhances PDAC cell proliferation and tumor growth in mice.

## Discussion

PDAC is one of the most lethal malignancies largely due to its extensive metabolic reprogramming and near-universal KRAS mutations (20). Although ketone body utilization has emerged as a critical metabolic dependency in PDAC, the regulatory logic that links dominant oncogenic signals to ketolytic machinery has remained elusive. In this study, we establish that oncogenic KRAS and IGF1 signaling act in concert to activate a previously unrecognized SUCLA2–OXCT1 axis, which drives hyperactive ketolysis to support energy production and tumor progression. Our work provides several conceptual and translational advances.

We identify ERK-mediated phosphorylation of SUCLA2 at S124 as a key molecular node that integrates KRAS and IGF1 signals (21, 22, 23). Notably, we observed isoform specificity in this regulation: ERK2, but not ERK1, phosphorylates SUCLA2. This specificity, consistent with reports that ERK1 and ERK2 have distinct substrate preferences despite high sequence homology, may have implications for the selective regulation of mitochondrial metabolic enzymes. While IGF1 signaling is known to sustain PDAC growth and KRAS is the primary oncogenic driver (21, 22, 23), our study demonstrates that these two pathways converge on the mitochondrial metabolic enzyme SUCLA2. By showing that ERK directly phosphorylates SUCLA2 to promote its interaction with OXCT1, we reveal a direct signaling cascade connecting membrane/nuclear oncogenic pathways to mitochondrial ketone metabolism, which extends the canonical view of KRAS effector signaling beyond proliferation and survival. Then we define PIN1-mediated cis–trans isomerization as an essential checkpoint for SUCLA2–OXCT1 complex assembly. PIN1 has been implicated in cancer progression by regulating oncogenic transcription factors and kinases; however, its role in controlling metabolic enzyme assembly in mitochondria is largely underappreciated. Our results demonstrate that phosphorylation of SUCLA2 creates a PIN1-binding motif, and PIN1-catalyzed conformational rearrangement is required for efficient SUCLA2–OXCT1 binding. This finding adds a critical conformational regulatory layer to metabolic reprogramming and highlights PIN1 as a potential upstream target to disrupt ketolysis in PDAC.

We demonstrate that SUCLA2 acts as a *bona fide* regulator of OXCT1 succinylation at K421, which represents a key post-translational mechanism governing ketolysis. Although OXCT1 is known as the rate-limiting enzyme for ketone breakdown (24, 25), its enzymatic activation by post-translational modification has not been defined in PDAC. Our data show that SUCLA2 not only produces succinyl-CoA as a ketolytic substrate but also directs site-specific succinylation to activate OXCT1. This dual function of SUCLA2—serving as both a metabolic enzyme and a signaling adaptor—reveals a new paradigm of metabolic enzyme cross-talk in cancer.

The steady-state level of OXCT1 succinylation is determined not only by the succinyltransferase activity of SUCLA2 but also by the opposing action of desuccinylases. Based on the mitochondrial matrix localization of OXCT1 and published evidence that SIRT5 is the principal NAD^+^-dependent lysine desuccinylase in this compartment (26), we hypothesized that SIRT5 might reverse OXCT1 succinylation. Pharmacological modulation of desuccinylase activity—using a SIRT5 inhibitor and a desuccinylase activator—yielded opposing effects on OXCT1 succinylation, consistent with the model that SIRT5 acts as a desuccinylase for OXCT1. These pharmacological data strongly support a role for SIRT5 in regulating OXCT1 succinylation dynamics. Complementary genetic approaches, such as SIRT5 knockout or overexpression, could further delineate the functional consequences of this regulation. Additionally, while our findings point to SIRT5 as a key desuccinylase, we cannot exclude the possibility that other mitochondrial desuccinylases (*e*.*g*., SIRT3 or SIRT4) may also contribute, either redundantly or in a context-dependent manner. Future studies aimed at dissecting the interplay among these enzymes will provide a more comprehensive view of the desuccinylation machinery governing OXCT1.

Our study uncovers a synthetic pro-tumorigenic interaction between KRAS mutation and IGF1 signaling in driving ketone body metabolism. Notably, KRAS mutation alone or IGF1 alone is insufficient to trigger maximal ketolytic flux and cell proliferation; instead, the two signals cooperate to achieve robust activation of the SUCLA2–OXCT1 pathway. This synergistic model is highly consistent with the *in vivo* context of PDAC, in which tumor cells harboring KRAS mutations are exposed to persistent IGF1 signaling. Thus, our findings help explain why PDAC cells develop a strong dependency on ketone bodies under nutrient stress.

Clinically, we show that SUCLA2 S124 phosphorylation is markedly elevated in KRAS-mutant human PDAC tissues, supporting the pathophysiological relevance of this axis. Given that KRAS mutations are present in >90% of PDAC patients, the SUCLA2–OXCT1 ketolytic axis may represent a targetable vulnerability specific to KRAS-driven pancreatic cancer. Therapeutically, small-molecule inhibitors targeting SUCLA2 phosphorylation, OXCT1 succinylation, or PIN1 may selectively block ketone body utilization and suppress tumor growth without disrupting normal tissue homeostasis.

Several limitations should be noted. First, the clinical sample size in this study is relatively small; future studies with larger cohorts are warranted to validate the prognostic value of SUCLA2 phosphorylation and OXCT1 succinylation. Second, the impact of this axis on the tumor microenvironment, including immune cell infiltration and stromal activation, remains to be explored. Third, further structural studies will be required to precisely delineate how succinylation at K421 allosterically enhances OXCT1 catalytic activity. Fourth, while our pharmacological data implicate SIRT5 as a candidate desuccinylase, genetic validation is needed to confirm its role.

In summary, our findings delineate a novel KRAS/IGF1–ERK–PIN1–SUCLA2–OXCT1 signaling cascade that reprograms ketone body metabolism to promote PDAC progression. This work not only expands our understanding of oncogenic signaling-controlled metabolic rewiring but also offers a promising therapeutic strategy for treating pancreatic cancer by targeting the SUCLA2–OXCT1 ketolytic axis.

## Experimental procedure

### Materials

Antibodies to SUCLA2 (12627-1-AP), OXCT1 (12175-1-AP), PIN1 (10495-1-AP), Flag-tag (66008-4-lg), His-tag (66005-1-lg), GST-tag (660012-lg) were purchased from Proteintech. The antibody to β-actin was purchased from Servicebio. Antibodies to ERK1/2 (ET1601-29), phospho-Erk1 (T202 + Y204) + Erk2 (T185 + Y187) (ET1610-13) were purchased from Huabio. Antibodies to normal mouse IgG (#2729), normal rabbit IgG (#68860) were purchased from Cell Signaling Technology. The antibody succinyl-lysine (#PTM-401, PTM-419) was purchased from PTM BIO. The antibody SUCLA2 pS124 and succinyl-CoA, β-HB were generous gifts from Dr. Dong Guo (Zhejiang University School of Medicine) (2). IGF1 was purchased from Sangon Biotech (Shanghai). β-Hydroxybutyric Acid (β-HB) Content Assay Kit (BC5085), ATP Content Assay Kit (BC5470), Acetoacetate (AcAc) Content Assay Kit (BC5070) and Acetyl CoA Content Assay Kit (BC0980) were purchased from Beijing Solarbio Science & Technology. Cell Counting Kit-8 (C0005) was purchased from TargetMol. All primary antibodies were validated by the manufacturer for specificity and were used according to the datasheet. In our hands, each antibody detected a single band at the expected molecular weight, and no signal was observed in the corresponding knockout/knockdown samples (where applicable).

### Cell culture

Cells were maintained in DMEM with 10% fetal bovine serum (Gibco). Cells were plated at a density of 5 × 10^6^ cells per 100-mm dish or 2 × 10^5^ cells per well in a six-well plate 12 h before transfection. No cell lines used in this study were found in the database of commonly misidentified cell lines maintained by the International Cell Line Authentication Committee and NCBI BioSample. All cell lines were authenticated by short tandem repeat (STR) profiling prior to the experiments and tested negative for *mycoplasma* contamination. Overnight serum starvation was performed before IGF1 and β-HB treatment.

### Co-immunoprecipitation and immunoblot analysis

Following cell lysis, the supernatant was collected by centrifugation. An aliquot of the supernatant was reserved as the Input control, while the remainder was pre-cleared with species-matched IgG. Specific antibodies were then added to form immunocomplexes, which were captured using Protein A/G beads. After a series of washes and elution, the supernatant was collected by centrifugation and subjected to Western blot analysis. The immunoprecipitated proteins were separated by SDS-polyacrylamide gel electrophoresis (SDS-PAGE) and transferred onto a PVDF membrane. The membrane was blocked, followed by sequential incubation with appropriate primary and species-matched secondary antibodies. After thorough washing, the signal was developed using an enhanced chemiluminescence (ECL) substrate.

### DNA constructs and mutagenesis

The plasmid encoding ERK2 was a gift from Dong Guo. SUCLA2, OXCT1, and PIN1 were amplified by PCR and cloned into pcDNA3.1/puro-Flag, pcDNA3.1/puro-HA, pLVX-puro-Flag, pLVX-puro-HA, pCold-His, and pGEX-4T-1–GST vectors, respectively. The shRNA target sequences were as follows: control shRNA, 5′-GCTTCTAACACCGGAGGTCTT-3′; OXCT1-shRNA, 5′-GGCAAGGAAACAGTTACTATT-3′; SUCLA2-shRNA, 5′-CCCAGGAGAGAATACTACTTT-3′; PIN1-shRNA5′-CCACCGTCACACAGTATTTAT-3′.

### Lentiviral production and infection

Lentiviruses were produced in HEK-293T cells. Briefly, HEK-293T cells were co-transfected with a lentiviral vector carrying either the gene of interest or a non-targeting control sequence, along with the psPAX2 and pMD2.G packaging plasmids. The culture medium was replaced 24 h post-transfection. Viral supernatants were collected between 48 and 72 h, followed by centrifugation to remove cellular debris. For infection, the viral supernatant was mixed with an equal volume of fresh medium and added to the target cells. After 12 h, the medium was replaced with fresh virus-containing medium. Puromycin selection or subsequent experiments were initiated 48 h post-infection.

### Purification of recombinant proteins

Protein expression and purification were performed as previously described (27). Briefly, the respective constructs were expressed in the *E*. *coli* BL21 strain. Bacterial cultures were grown at 37 °C until the optical density at 600 nm (OD_600_) reached 0.6. Protein expression was then induced with 0.5 mM isopropyl β-d-1-thiogalactopyranoside (IPTG) and carried out overnight at 16 °C. For GST-tagged proteins, the clarified lysate was loaded onto a GSTrap HP column. The column was washed with five column volumes of phosphate-buffered saline (PBS), and the target protein was subsequently eluted with 10 mM reduced glutathione. For His-tagged proteins, the clarified lysate was applied to a Ni-NTA column. After a wash step with five column volumes of 20 mM imidazole, the protein was eluted using 250 mM imidazole.

### GST pull-down assay

The purified GST-tagged protein was incubated with glutathione agarose beads at 4 °C overnight. The prey protein was then added to the mixture and further incubated to allow binding. After extensive washing and elution, the specifically bound proteins were analyzed and verified by Western blotting.

### *In vitro* succinylation assay

As described previously (2), an *in vitro* succinylation assay was performed by incubating bacterially purified His-OXCT1 protein with the indicated concentrations of succinyl-CoA in TBS buffer (50 mM Tris-HCl, pH 7.4, 150 mM NaCl) at 37 °C for 30 min.

### Cell proliferation assay

Cells were seeded in 96-well plates at a density of 1 × 10^4^ cells per well in 100 μl of complete culture medium. Following serum starvation, the cells were treated with or without 2 mM β-HB for 48 h in the presence of IGF1. Subsequently, 10 μl of CCK-8 solution was added to each well and incubated for 1 h. The absorbance was then measured at a wavelength of 450 nm using a microplate reader.

### Measurement of medium β-HB and intracellular AcAc, acetyl-CoA and ATP

The level of β-HB in the culture medium was quantified with a β-HB Assay Kit. Meanwhile, the intracellular levels of AcAc, acetyl-CoA, and ATP were determined using their specific assay kits, all in accordance with the manufacturers’ instructions.

### Animal studies

ASPC1 cells (5 × 10^5^ cells in 100 μl of PBS), with or without modulation of OXCT1 expression, were subcutaneously injected into 5-week-old male athymic nude mice (n = 5 per group). Subsequently, tumor tissues were harvested, fixed in 4% formaldehyde, and paraffin-embedded. Tumor volume was calculated using the formula V = a ∗ b^2^/2, where a and b represent the tumor length and width.

Subcutaneous tumor was dissected, fixed in 4% formaldehyde and embedded in paraffin for analysis. All animal procedures adhered to institutional and national guidelines and were approved by the institutional research ethics committee of Qingdao University School of Medicine. The maximal tumor burden permitted by the ethics committee (1500 mm^3^) was not exceeded in these experiments.

### *In vitro* kinase assay

As described previously (28). Briefly, the purified active recombinant GST-ERK2 protein (10 ng) was incubated with His-SUCLA2 protein (100 ng) in a 25 μl kinase reaction buffer containing 50 mM Tris-HCl (pH 7.5), 100 mM KCl, 50 mM MgCl_2_, 1 mM Na_3_VO_4_, 1 mM DTT, 5% glycerol, and 10 mM ATP. The reaction was carried out at 25 °C for 1 h and terminated by adding SDS-PAGE sample loading buffer followed by heating at 100 °C.

### Immunohistochemistry analysis

Human pancreatic tissue samples were obtained from patients who underwent surgical resection at The Affiliated Hospital of Qingdao University. The study was approved by the Human Ethics Committee of Qingdao University School of Medicine (approval No. QDU-HEC-2025564). Written informed consent was obtained from all patients prior to sample collection. The research was conducted in accordance with the Declaration of Helsinki. As previously described (17), paraffin-embedded sections from both mouse xenograft tumors and clinical human PDAC specimens were subjected to immunohistochemical staining with anti-Ki-67 or anti-SUCLA2 pS124 antibodies; non-specific rabbit IgG served as a negative control.

### Quantification and statistical analysis

Band intensities from Western blot analyses were quantified using ImageJ software. Unless otherwise specified, all data are presented as the mean ± standard deviation (SD) from three independent experiments or replicates. Comparisons between two groups were performed using an unpaired, two-tailed Student's *t* test. For comparisons across multiple groups and/or under multiple conditions, one-way or two-way analysis of variance (ANOVA) was applied. All statistical analyses were conducted using GraphPad Prism software, and a *p*-value of less than 0.05 was considered statistically significant.

## Data availability

All data generated or analyzed during this study are included in this published article and its supplementary information files.

## Conflict of interests

The authors declare the following financial interests/personal relationships which may be considered as potential competing interests:

Z. L. owns shares in Signalway Biotechnology (Pearland, TX), which supplied rabbit antibodies that recognize SUCLA2 pS124. Z. L.’s interest in this company had no bearing on its being chosen to supply these reagents. The other authors declare no competing interests.
